# Unveiling potential drug targets for lung squamous cell carcinoma through the integration of druggable genome and genome-wide association data

**DOI:** 10.3389/fgene.2024.1431684

**Published:** 2024-08-08

**Authors:** Wenhua Wu, Zhengrui Chen, Haiteng Wen, Haiyun Zhang

**Affiliations:** ^1^ The Second Clinical Medical College, Zhujiang Hospital, Southern Medical University, Guangzhou, China; ^2^ Department of Pulmonary and Critical Care Medicine, Zhujiang Hosptial, Southern Medical University, Guangzhou, Guangdong, China

**Keywords:** lung squamous cell carcinoma, drug targets, summary-data-based mendelian randomization, genomics, genome-wide association study

## Abstract

**Background:** Lung squamous cell carcinoma (LSCC) is a major subtype of lung cancer with poor prognosis and low survival rate. Compared with lung adenocarcinoma, yet no FDA-approved targeted-therapy has been found for lung squamous cell carcinoma.

**Methods:** To identify potential drug targets for LSCC, Summary-data-based Mendelian randomization (SMR) analysis was used to examine the potential association between 4,543 druggable genes and LSCC, followed by colocalization analysis and HEIDI tests to confirm the robustness of the result. Phenome-wide association study (PheWAS) explored potential side effects of candidate drug targets. Enrichment analysis and protein-protein interaction networks revealed the function and significance of therapeutic targets. Single-cell expression analysis was used to examine cell types with enrichment expression of druggable genes in LSCC tissue. Drug prediction included screening potential drug candidates and evaluating their interactions with targets through molecular docking.

**Results:** This research has identified ten significant drug targets for LSCC through a comprehensive SMR analysis. These targets included (COPA, PKD2L1, CCR1, C2, CYP21A2, and NCSTN as risk factors, and CCNA2, C4A, APOM, and LPAR2 as protective factors). PheWAS demonstrated that C2, CCNA2, LPAR2, and NCSTN exhibited associations with other phenotypes at the genetic level. Then, we found four potentially effective drugs with the Dsigdb database. Subsequently, molecular docking indicated that favorable binding interactions between drug candidates and potential target molecules. In the druggability evaluation, five out of ten drug target genes have been used in drug development (APOM, C4A, CCNA2, COPA, and PKD2L1). Six out of ten druggable genes showed significant expression in LSCC tissues (COPA, PKD2L1, CCR1, C2, NCSTN, LPAR2). Besides, Single-cell expression analysis revealed that C2 and CCNA2 were primarily enriched in macrophages, while COPA and NCSTN were enriched in both macrophages and epithelial cells.

**Conclusion:** Our research revealed ten potential druggable genes for LSCC treatment, which might help to advance the precise and efficient therapeutic approaches of LSCC.

## 1 Introduction

Lung cancer accounts for the highest proportion of total cancer cases (11.6%) and remains the primary cause of mortality associated with cancer (18.4%) (Bray et al., 2018). Traditionally, lung cancer is categorized into two main subtypes: small cell lung cancer (SCLC) and non-small cell lung cancer (NSCLC). NSCLC constitutes the majority (80%) of all lung cancers, with lung squamous cell cancer (LSCC) accounting for 20%–30% within this subgroup (Barta et al., 2019). In contrast to lung adenocarcinoma, targeted therapy offers limited benefits for patients with lung squamous cell carcinoma (Lau et al., 2022). The utilization of targeted therapy in patients with LSCC has been associated with unfavorable outcomes in previous investigations (Niu et al., 2022). Hence, there is necessary to find novel therapeutic targets to facilitate the development of LSCC targeted therapy.

The increasingly abundant human genetic data is now extensively utilized to explore innovative drugs for various diseases (Trajanoska et al., 2023). It is a useful way to find drug targets to improve the treatments of diseases through analyzing human genetic data (Nelson et al., 2015). Mendelian randomization (MR) is a genetics-based statistical approach that enables the assessment of causal relationships between modifiable exposure or risk factors and clinically relevant outcomes (Sekula et al., 2016). The Summary-based MR (SMR) analysis simulates randomized controlled trials by integrating aggregated data from disease genome-wide association studies (GWAS) and expression quantitative trait locus (eQTL) studies, enabling the prediction of drug efficacy (Cao et al., 2023). In the analysis of drug target MR, cis-expressed quantitative trait loci (cis-eQTLs) located within the genomic region of the drug target gene are commonly regarded as proxies that function as regulatory factors influencing gene expressions (Gaziano et al., 2021).

This study identified potential drug targets associated with LSCC by multi-omic analysis. First, SMR analysis was employed to investigated the potential association between druggable genes and LSCC. Since SMR alone may not be adequate for identication of reliable drug targets, additional colocalization analysis and heterogeneity in dependent instruments (HEIDI) tests were performed. These analyses further established a causal link between therapeutic targets and LSCC while mitigating potential confounding variables. The enriched cell types in LSCC tissues were determined through single-cell type expression analysis. Furthermore, our phenome-wide association study (PheWAS) delves into the relationships between potential therapeutic targets and additional characteristics, offering significant insights for future research and the formulation of pertinent therapeutic approaches. Subsequent enrichment analysis and the construction of a protein-protein interaction (PPI) network unveiled functional attributes and biological associations of potential therapeutic targets, enhancing our comprehensions of the mechanisms in the development and treatment of LSCC. Ultimately, drug prediction was conducted for the identified targets, followed by screening of potential drug candidates and assessment of their binding affinity and interaction mode with the targets using molecular docking.

## 2 Methods and materials

**FIGURE 1:**
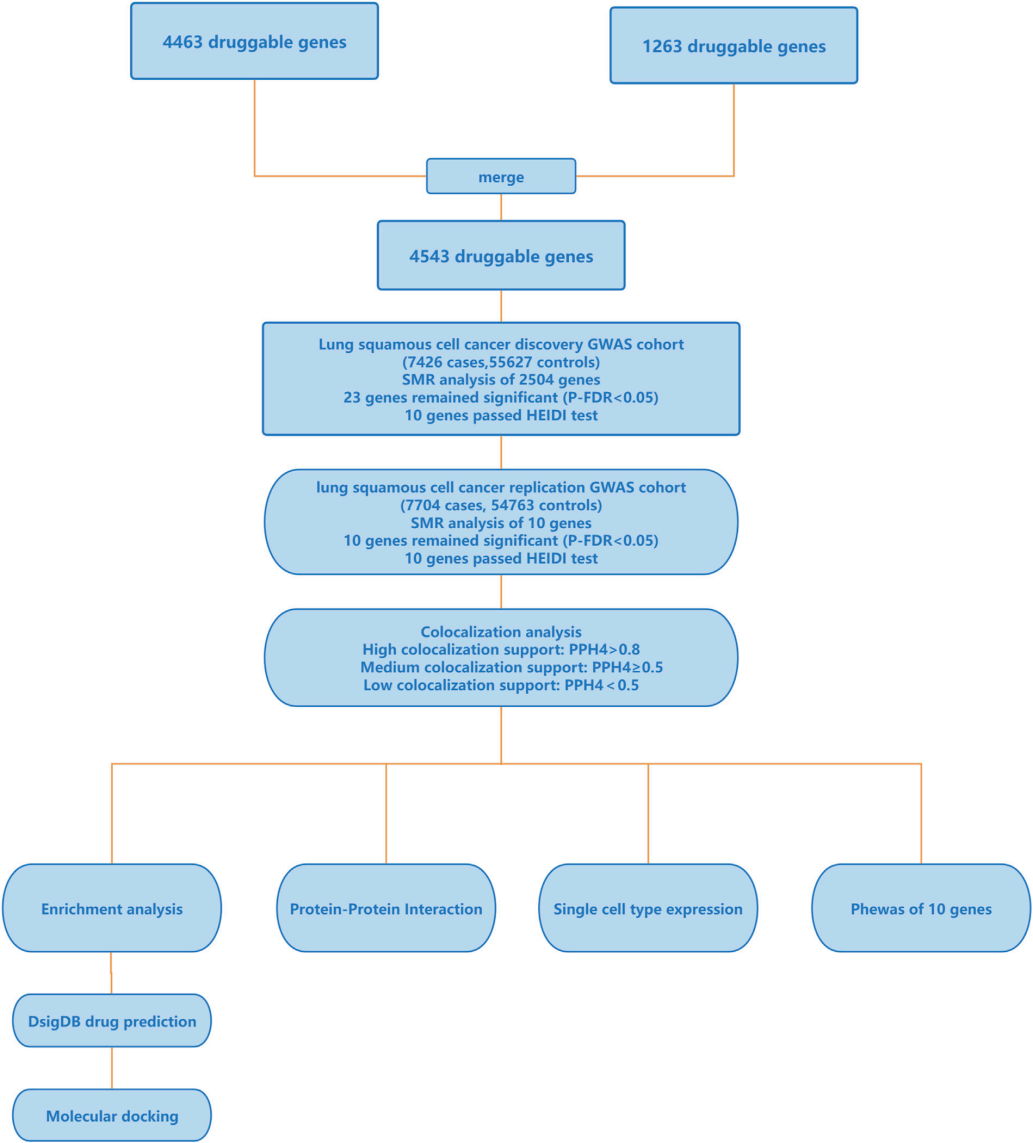
Outline of the study design. GWAS, genome-wide association studies; SMR, summary-data-based Mendelian Randomization; HEIDI, heterogeneity in dependent instruments; PPH4, the posterior probability of hypothesis 4; PheWAS, Phenome-Wide Association Study.

The flowchart of the study is depicted in Figure 1, and more information of the methods and materials are presented below. All data used in this research were sourced from publicly available databases, so no additional ethical review was necessary.

### 2.1 Data source for drug targets

The druggable genes selected for this study were identified based on a recent research on druggable genome conducted by Finan *et al*. (Finan et al., 2017), as well as the inclusion of 1,263 actionable drug targets provided by Gaziano *et al* (Gaziano et al., 2021)*.* Finan *et al.*’s study developed a new computational approach and integrated data from multiple GWAS to reveal druggable proteins and link them to existing drugs, resulting in 4,463 genes with potential for therapeutic targets. To identify potential drug targets against COVID-19, Gaziano *et al.* collected 1,263 druggable proteins from the ChEMBL database. Among these proteins, 531 have been validated as therapeutic targets for approved drugs, while 381 were in clinical trials and 351 proteins exhibited promising potential as targets for approved medications. For more detailed information on the aforementioned druggable genes, please refer to the original publication and Supplementary Material (Supplementary Tables S2, S3).

### 2.2 Exposure data

We merged two list of drug targets, resulting in a total of 4,543 druggable genes named by the Human Genome Organization Gene Nomenclature Committee (Supplementary Table S4). Given that cis-eQTL demonstrated a higher degree of proximity to genes that possess potential for drug target in the drug development research, we selected cis-eQTL linked with gene expression within 2000 kb of the corresponding available druggable genes from the eQTLGen Consortium (Vosa et al., 2021). The eQTLGen Consortium contains cis-eQTLs of 16,987 genes sourced from 31,684 individuals of healthy European ancestry. We employed the standard threshold of 5e-8 for genome-wide significant *p*-value to identify the most significant eQTL as instrumental variables in SMR analysis. In the final, we identified eQTLs for 2,504 druggable genes.

### 2.3 Outcome data

In the discovery cohort, the GWAS data for LSCC was obtained from a meta-analysis conducted by McKay et al. (McKay et al., 2017). This meta-analysis involved a total of 7,426 patients and 55,627 controls. Additionally, the GWAS data for LSCC from the Interdisciplinary Research in Lung Cancer (TRICL) consortium, which consisted of 7,704 patients and 54,763 controls, was utilized as the replication cohort. The patients of the discovery and replication cohorts were diagnosed with squamous cell lung carcinoma according to the histopathological and immunohistochemical methods. More details about the eQTL and GWAS data are available in Supplementary Table S1 **.**

### 2.4 SMR analysis

SMR analysis was used to evaluate the pleiotropic association between druggable genes expression and squamous cell lung cancer, as it has higher statistical power than traditional two-sample MR analysis when data from two independent populations with large sample sizes are available (Zhu et al., 2016). To further examine the heterogeneity in causal inference, we employed the HEIDI test and excluded results with P_-HEIDI_ < 0.05 (Zhu et al., 2016). The SMR software tool (version 1.3.1) was used to perform SMR and HEIDI tests (Wu et al., 2018). To address the bias from multiple test we adjusted the *p*-value using the Benjamini–Hochberg method to control for a false discovery rate (FDR) of 0.05 (Korthauer et al.). We then selected genes with FDR of *p*-value <0.05 and P_-HEIDI_ > 0.05 in replication cohorts for further co-localization analysis.

### 2.5 Colocalization analysis

To determine whether druggable genes and LSCC shared the same genetic variant, we used Bayesian colocalization analysis using eQTL and LSCC GWAS summary statistics with coloc R package (Giambartolomei et al., 2014). Colocalization analysis consists of five exclusive hypotheses (H0-H4): H0, no causal variants are associated with either traits; H1, a causal variant associated with gene expression but not with LSCC risk; H2, a causal variant associated with LSCC risk but not with gene expression; H3, associated with LSCC risk and gene expression but driven by distinct causal variants; H4, associated with LSCC risk and gene expression, driven by the same genetic variation. The degree of colocalization was quantified by posterior probability of hypothesis 4 (PPH4). A PPH4 value exceeding 0.8 suggests strong co-localization support, whereas values falling within the range of 0.5–0.8 suggests moderate co-localization support, with PPH4 values of 0.5 or lower suggesting weak co-localization support.

### 2.6 Phenome-wide association study

In this research, the AstraZeneca PheWAS Portal (https://azphewas.com/) was employed to conduct PheWAS analysis, aiming to deduce potential side effects of prospective drug targets. The PheWAS used data from United Kingdom Biobank, including around 15.5 k binary phenotypes and 1.5 k continuous phenotypic data from about 450,000 exome sequencing participants (Wang et al., 2021). A *p*-value < 1e-6 was considered significant for PheWAS.

### 2.7 Enrichment analysis and protein-protein interaction

Gene ontology (GO) and Kyoto Encyclopedia of Genes and Genomes (KEGG) pathway enrichment analysis of these ten drug target genes were performed to explore their potential biological functions and pathways using R package clusterprofiler. GO was categorized into three groups: biological process (BP), molecular function (MF), and cellular component (CC). KEGG can provide information for signaling pathway. In addition, a PPI network was generated by utilizing the STRING database (https://string-db.org/) to explore potential interactions among ten drug target genes. The obtained outcome was then imported into Cytoscape for visualization.

### 2.8 Single cell type expression analysis

To investigate the cell-population specific expression of target genes and study their potential causal effect on LSCC, scRNA-seq data for human LSCC tissues were further obtained from Zilionis R et al. (Zilionis et al., 2019). The datasets were deposited in the Gene Expression Omnibus (GEO) database (GSE127465). Purified LSCC samples in batches were firstly integrated and corrected using “IntegrateData” function. Quality control standards are as follows: 500 < nFeature_RNA < 5,000; 200 < nCount_RNA < 35,000; and percentage. mt < 10%. Then, annotation of cell clusters was carried out using “SingleR” package and CellMarker databases (Aran et al., 2019).

### 2.9 Candidate drug prediction and druggability prediction

We used DSigDB database to further investigate whether the identified drug target genes can become potential effective intervention drugs by studying the interactions between these proteins and drugs (Yoo et al., 2015). In order to further study the drug potential of existing drug target genes, we used PDB, ChEMBL and DrugBank databases (Berman et al., 2000; Wishart et al., 2018; Mendez et al., 2019), which containing comprehensive molecular information about drugs, their mechanisms, their interactions and their targets. We then collected the information on drug names and the development process of drugs that targeted identified proteins.

### 2.10 Molecular docking

The 3D structure (.pdb format file) of the core common target was downloaded using the PDB database (https://www.rcsb.org/), and the small molecule drug structure (sdf format file) was collected using the PubChem database (https://pubchem. ncbi. nlm.nih.gov/). Molecular docking was performed by online docking tool CB-Dock2 (Liu et al., 2022). The ligand molecule can spontaneously bind to the receptor protein when its binding energy is less than 0, whereas a lower binding energy indicates a tighter binding between the two. The specific parameter setting principle is based on the coordinate corresponding to the compound originally bound on the target pocket of the protein as the center, so as to carry out lattice construction.

## 3 Result

### 3.1 SMR analysis and HEIDI test found 10 druggable genes associated with LSCC

**FIGURE 2:**
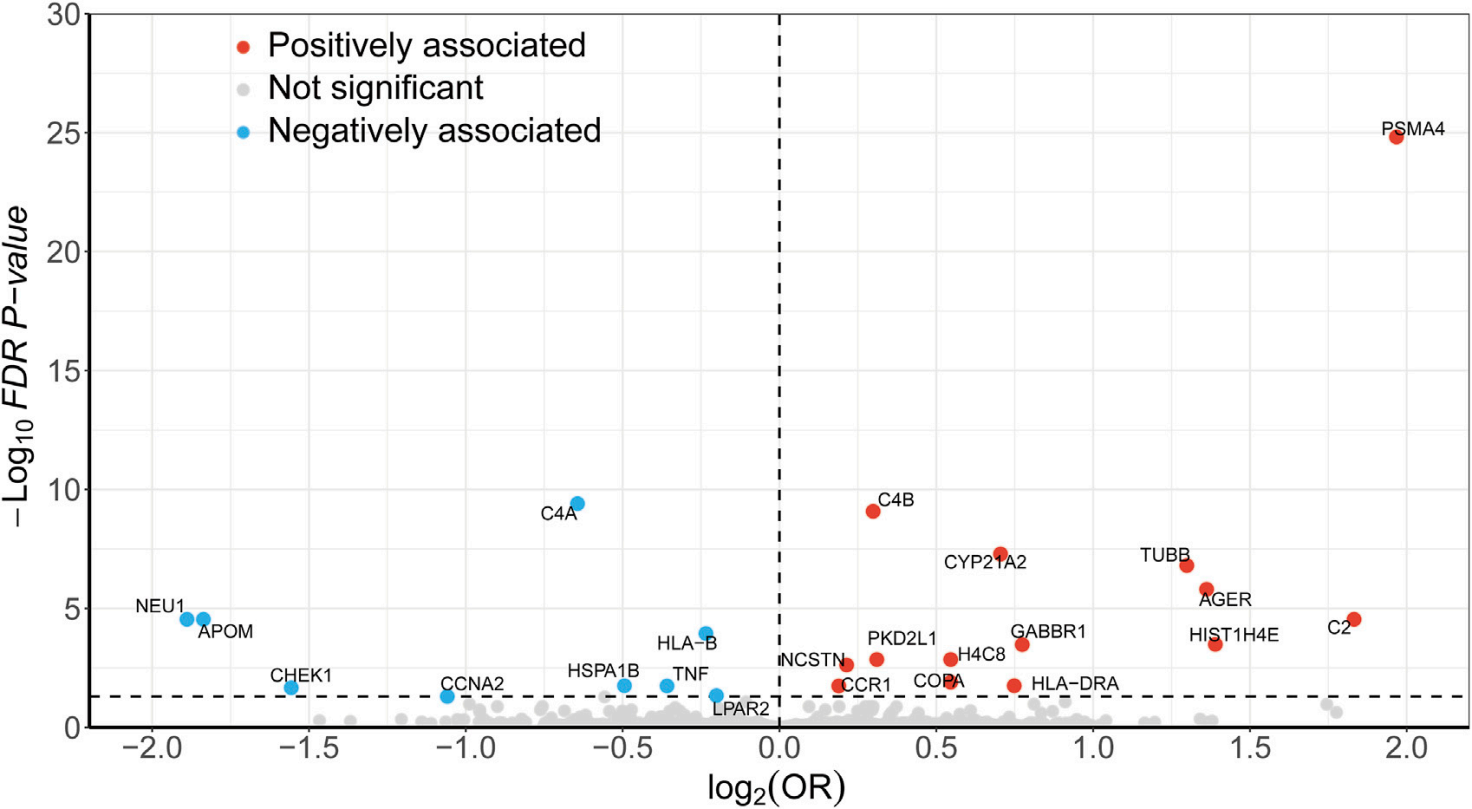
Volcano plot displaying the SMR results from the discovery phase for 23 significant genes. Dashed line on the horizontal axis represents FDR 0.05.

**FIGURE 3:**
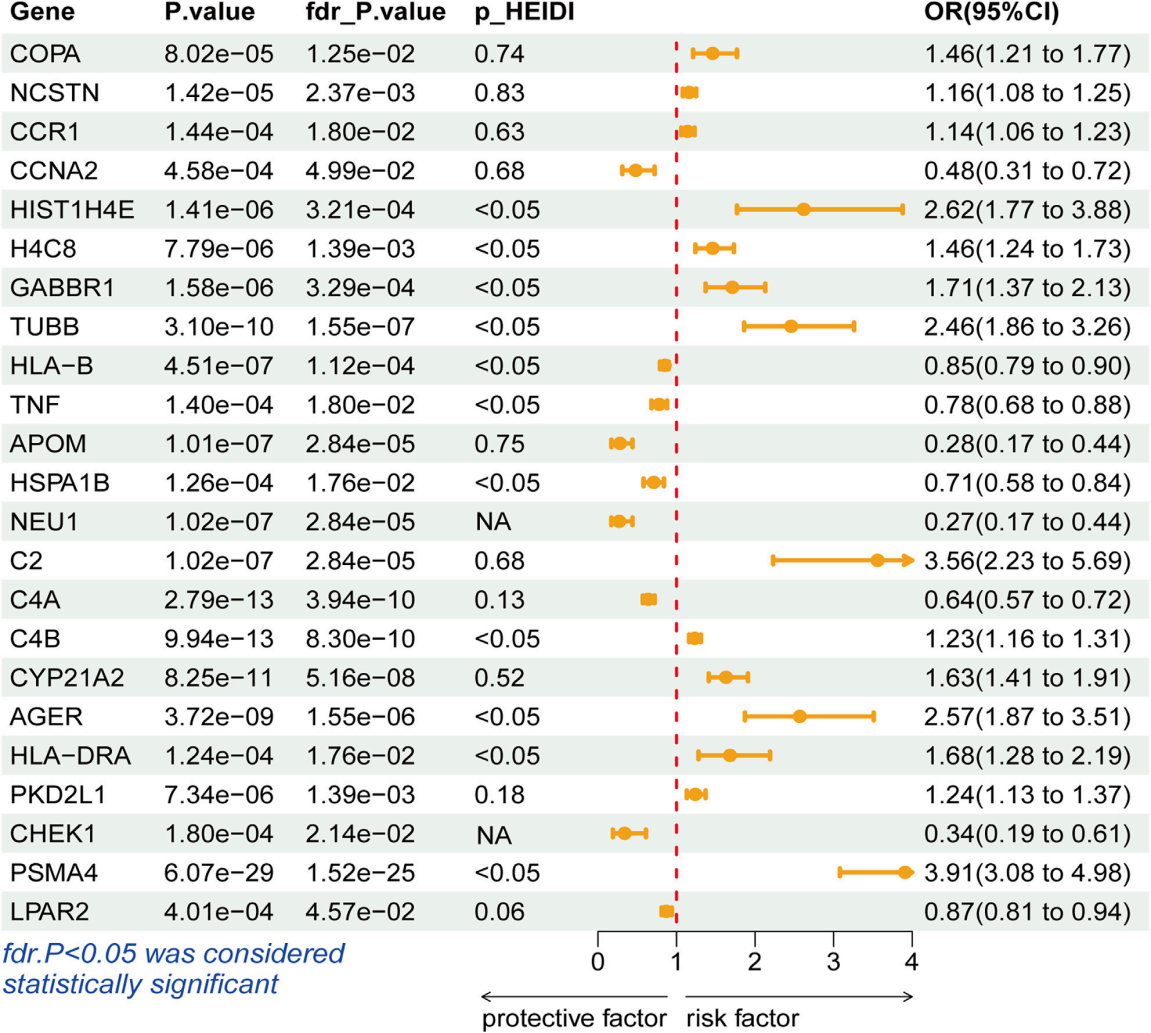
Forest plots displaying the SMR results from the discovery phase for 23 significant genes.

During the discovery stage, the expression of 23 genes was identified significantly linked with the risk of LSCC (P-FDR < 0.05), as illustrated in Figure 2. However, after conducting the HEIDI test, 13 of these genes did not meet the required criteria (P_-HEIDI_ < 0.05) (Figure 3). Therefore, they were excluded from subsequent replication analyses (Supplementary Table S5). In the final, we obtained 10 druggable genes for further analysis.

**FIGURE 4:**
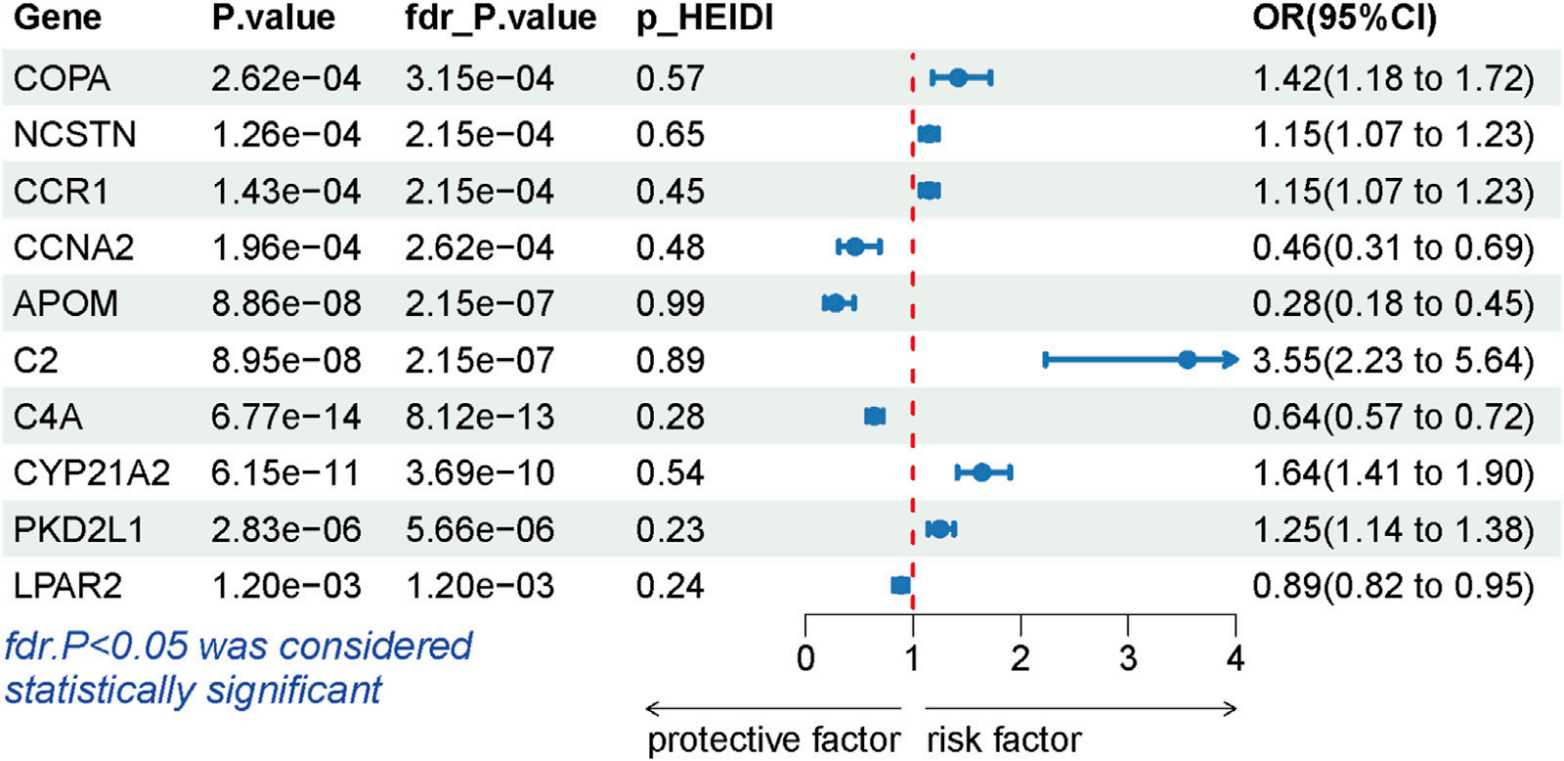
Forest plots displaying the SMR results from the replication phase for 10 significant genes.

During the replication stage, SMR analysis successfully validated 10 genes for another independent LSCC cohort. The expression of 10 genes still exhibited a significant association with the risk of LSCC (P-FDR <0.05), as illustrated in Figure 4. Furthermore, all of these genes successfully passed the HEIDI test, thereby indicating the robustness of the results (Supplementary Table S6).

### 3.2 Colocalization analysis

The results of the colocalization analysis were presented in Supplementary Table S7. Among the 10 potential drug targets, COPA, CCNA2, PKD2L1 shown high support evidence of colocalization. Four genes (CCR1, C2, C4A, and CYP21A2) demonstrated medium support evidence of colocalization. The remaining three genes including NCST, APOM, and LPAR2, indicated low support evidence of colocalization.

### 3.3 PheWAS

The PheWAS findings can provide information on the correlation between identified drug target gene expression and certain diseases or traits. In Supplementary Figures S1−S12, at the gene level, there was no significant association between five drug targets and other traits (genomic association *p*-value < 1e-6), with the exception of CCNA2, C2, and NCSTN, while C4A was not found in the database. CCNA2 was associated with factors influencing health status and contact with health services while C2 was associated with proteomics in cardiometabolic. NCSTN was associated with diseases of the skin and subcutaneous tissue. Additionally, LPAR2 was associated with lipoprotein metabolism and oncology at the variant level. The correlation between the above three genes and other phenotypes were shown in Supplementary Table S8, suggesting that the lung squamous cell carcinoma drugs acting on the three genes may affect these traits at the same time while the MR results of the three genes may have a pleiotropic effect.

### 3.4 Enrichment analysis and PPI network

**FIGURE 5:**
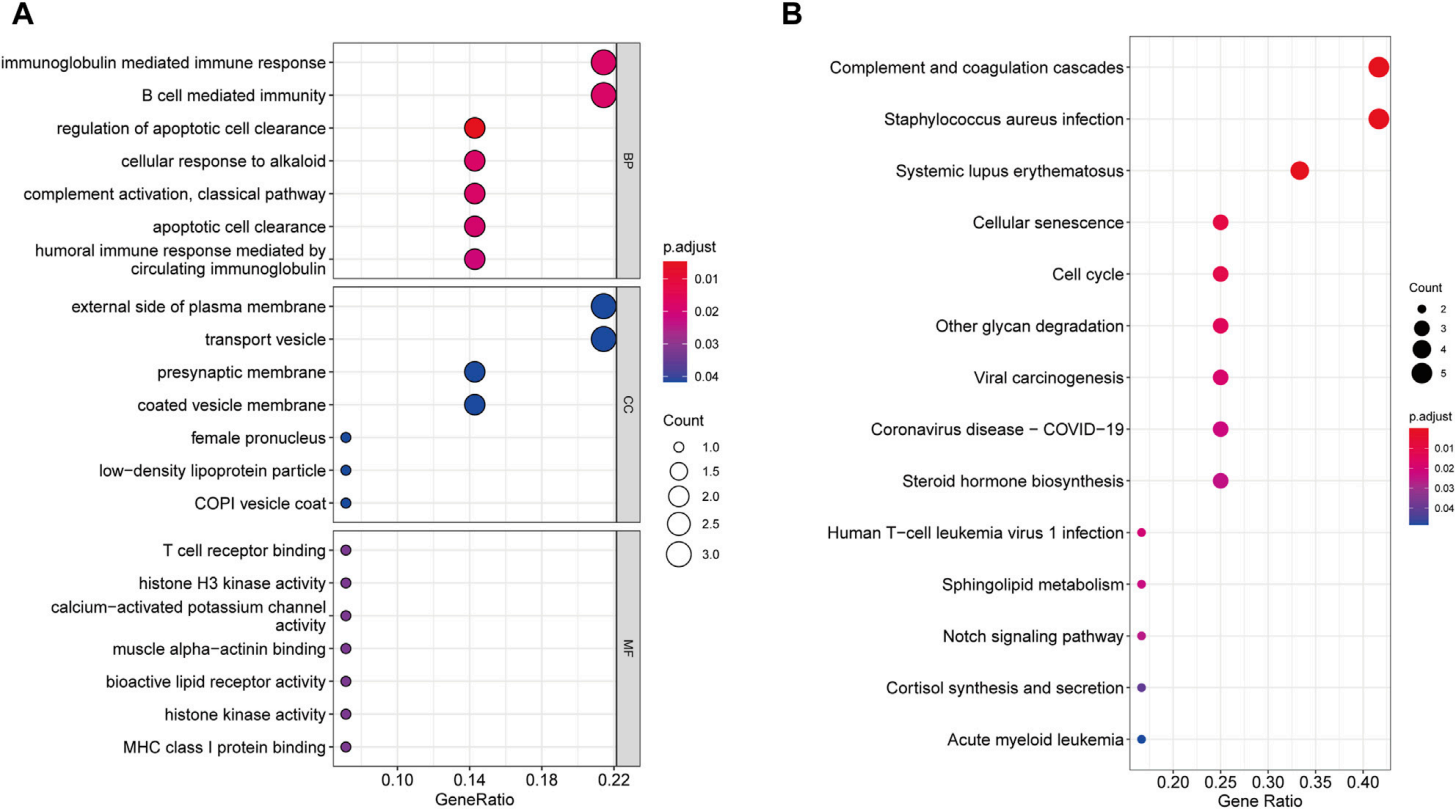
Enrichment results of 10 drug target genes. **(A)** Go enrichment results. **(B)** KEGG enrichment results.

**FIGURE 6:**
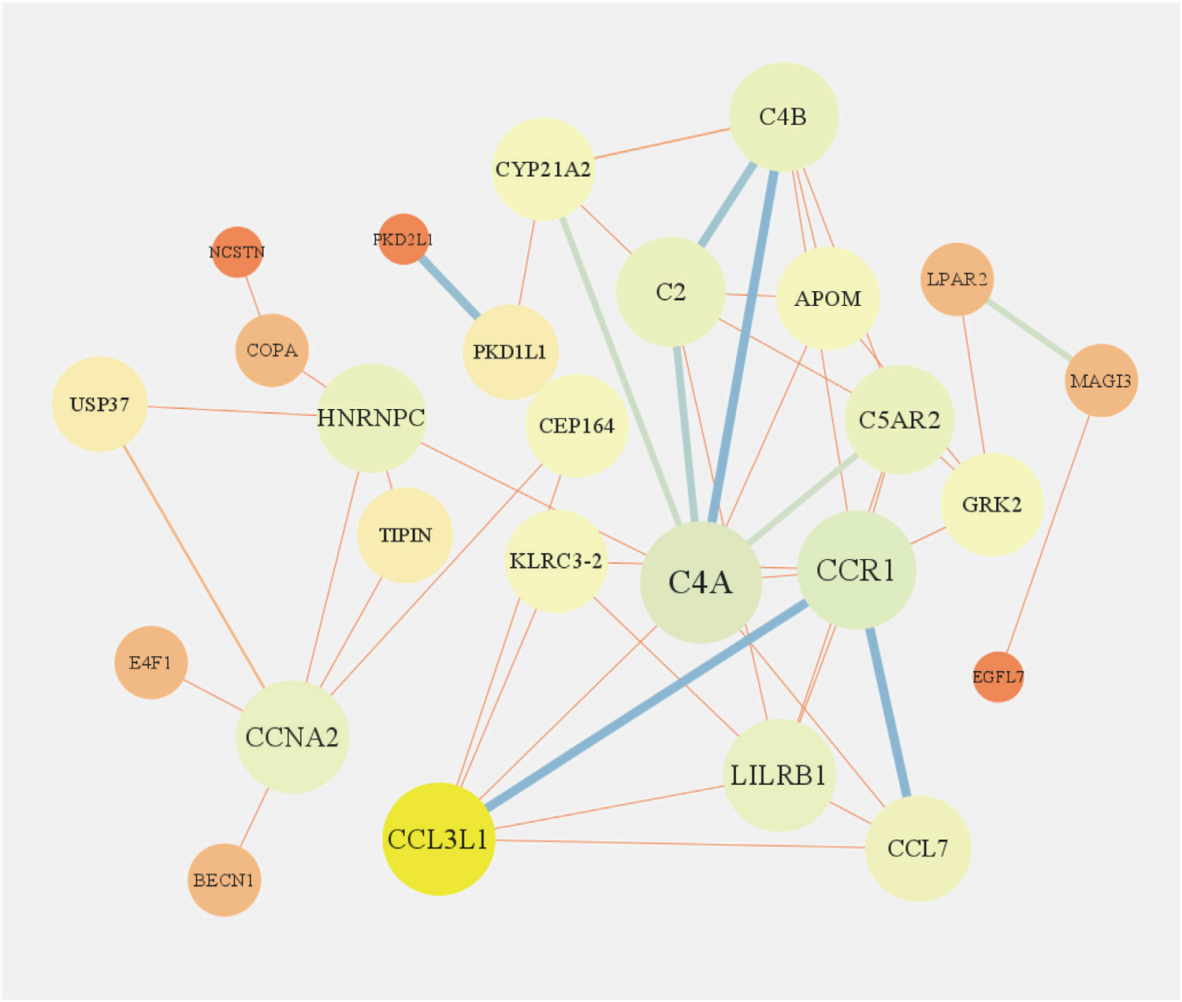
PPI network.

According to GO enrichment analysis, the druggable genes were enriched in the BP of immunoglobulin mediated immune response and B cell mediated immunity, and were related to the CC category of external side of plasma membrane and transport vesicle. In class MF, those target drug genes were enriched in T cell receptor binding and histone H3 kinase activity (Figure 5A). As shown in Figure 5B, KEGG enrichment analysis revealed that target drug genes were involved in complement and coagulation cascades, *staphylococcus aureus* infection, systemic lupus erythematosus, cellular senescence and cell cycle. The infection caused by *Staphylococcus aureus* can result in extensive inflammation of the dermis and subcutaneous tissue, while systemic lupus erythematosus is an autoimmune disease. Figure 6 indicates that the ten drug targets interact with other related proteins in a network with 29-node, 63-edge. C4A was significantly associated with a variety of proteins including CYP21A2, APOM, and C2.

### 3.5 Druggability evaluation on the potentials of therapeutic targets

Using the Dsigdb database, Table 1 shows the top four potential effective intervention drugs with threshold adjusted *p* < 0.05. The results showed that CCNA2 was significantly associated with most drugs, including ciglitazone (CTD 00001835), simvastatin (CTD 00007319) and irinotecan hydrochloride (CTD 00002224). In addition, irinotecan hydrochloride (CTD 00002224) interacts with most genes, indicating its potential serving as an effective drug. In druggability evaluation, we found that five of the ten drug target genes have been used for drug development (APOM, C4A, CCNA2, COPA, and PKD2L1) (Supplementary Table S9). Among them, drugs developed for C4A, COPA, and PKD2L1 targets have been approved. Drug (Human immunoglobulin G) targeting C4A is used in the treatment of immunodeficiencies, as well as autoimmune and inflammatory disorders. Drug (Artenimol) targeting COPA is an artemisinin derivative and antimalarial agent used in the treatment of uncomplicated plasmodium falciparum infections. Drug (Calcium citrate) targeting PKD2L1 is an ingredient found in a variety of supplements and vitamins.

**TABLE 1:** Candidate drug predicted using DSigDB.

Drug names	Overlap	*p*-Value	Adjusted *p*-value	Genes
Isotretinoin PC3 UP	2/24	6.17E-05	0.01	C4A; CYP21A2
ciglitazone CTD 00001835	3/190	9.64E-05	0.01	CCNA2; COPA; APOM
Irinotecan hydrochloride CTD 00002224	4/565	0	0.01	CCR1; CCNA2; COPA; NCSTN
simvastatin CTD 00007319	3/304	0	0.03	CCR1; CCNA2; APOM

### 3.6 Cell-type specificity expression in the LSCC tissues

**FIGURE 7:**
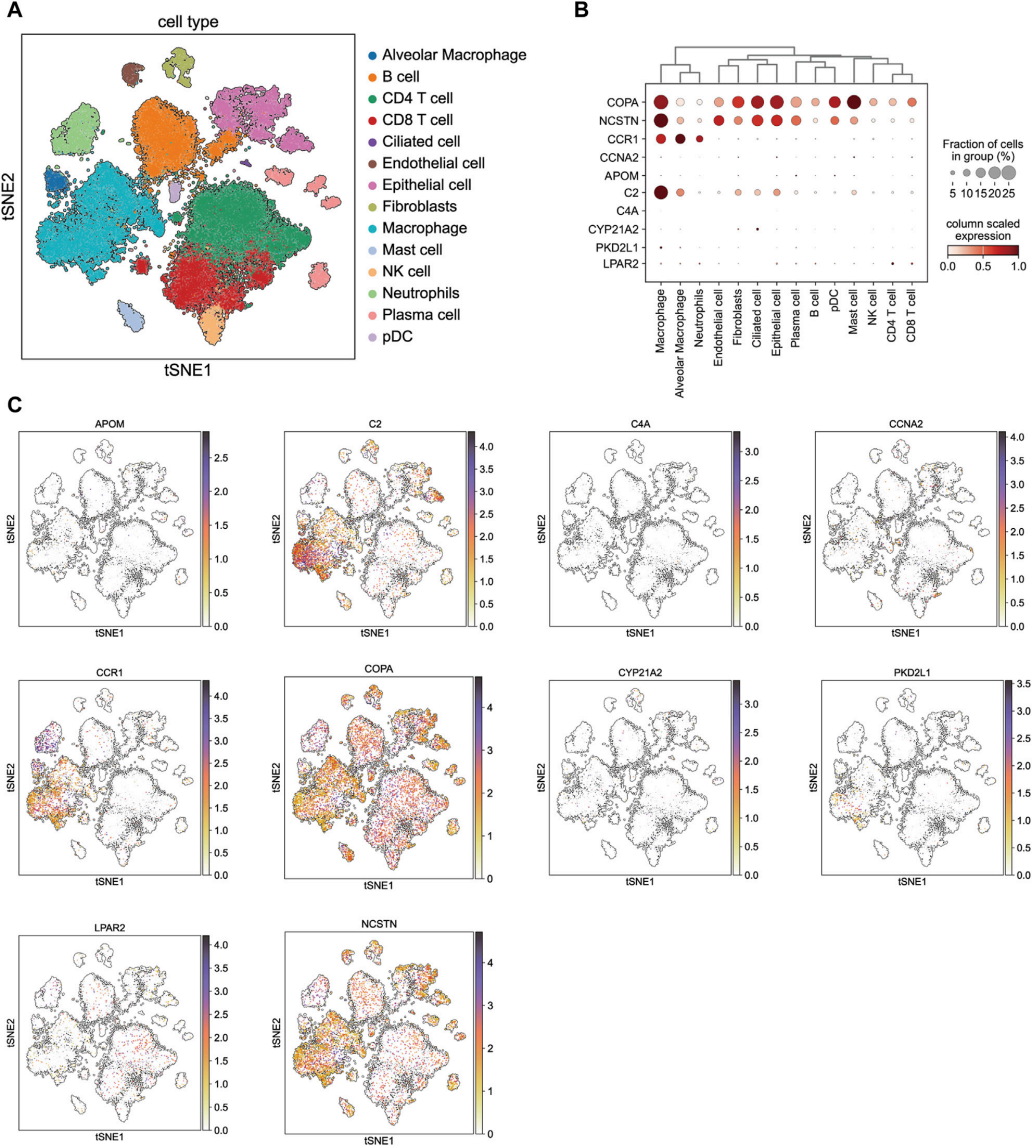
Single-cell type expression in lung squamous cell carcinoma tissue for the drug target genes identified by SMR. **(A)** 22 cell clusters and 14 cell types were identified. **(B)** and **(C)** show the expression of drug target genes in each cluster.

In order to further explore whether there were cell type-specific enrichment of 10 drug target genes, we applied single-cell RNA-seq data to perform single-cell expression analysis in lung squamous cell carcinoma tissues. All cells were divided into 22 clusters, and were identified into 14 major cellular subsets: alveolar macrophage, B cell, CD4^+^ T cell, CD8^+^ T cell, ciliated cell, endothelial cell, epithelial cell, fibroblasts, macrophage, mast cell, NK cell, neutrophils, plasma cell and pDC (Figure 7A). The single-cell expression of these genes is presented as a bubble chart in Figure 7B. Five of the ten target drug genes are detected in lung squamous cell carcinoma tissues, whereas the expression of CCNA2, APOM, C4A, PKD2L1, and CYP21A2 was not very significant. Notably, C2 and CCR1 were primarily enriched in the macrophage population (Figure 7C), while COPA and NCSTN were mainly enriched in both macrophage and epithelial cells.

### 3.7 Molecular docking

**FIGURE 8:**
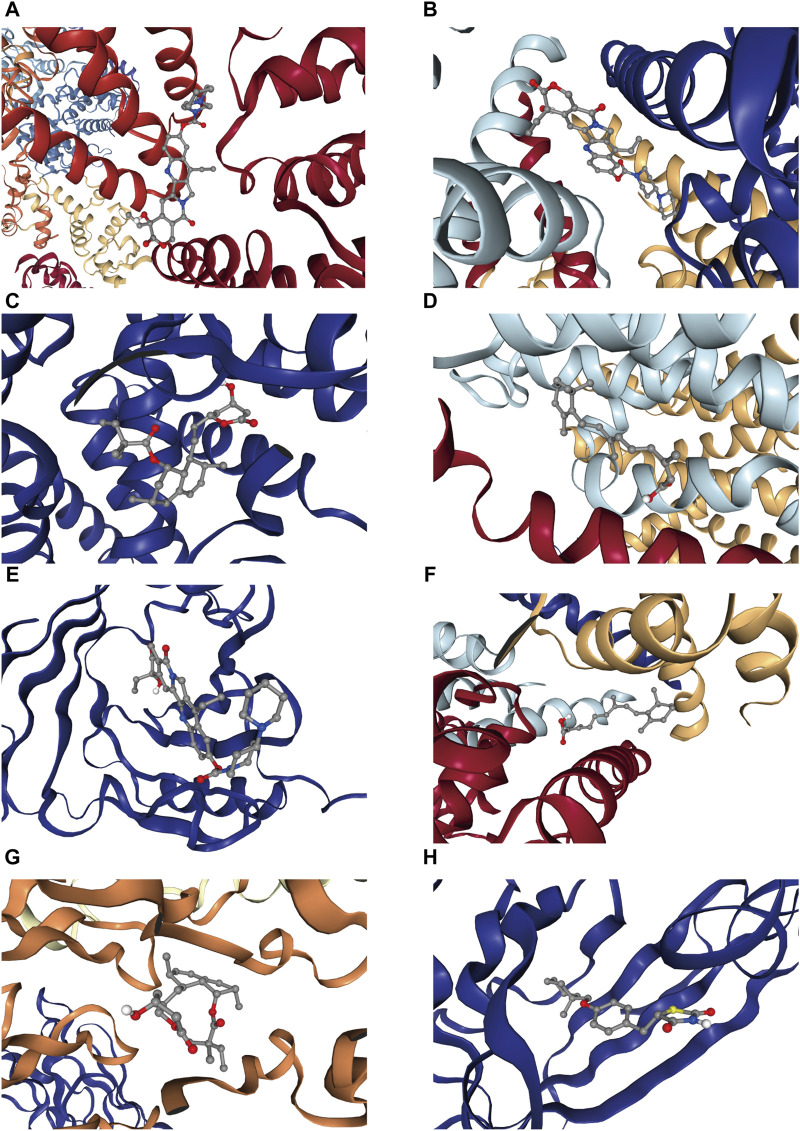
Docking results of available proteins with small molecule ligands. **(A)** CCNA2 docking Irinotecan hydrochloride, **(B)** PKD2L1 docking Irinotecan hydrochloride, **(C)** CYP21A2 docking simvastatin, **(D)** NCSTN docking isotretinoin, **(E)** APOM docking Irinotecan hydrochloride, **(F)** PKD2L1 docking isotretinoin, **(G)** CCR1 docking simvastatin, **(H)** APOM docking ciglitazone.

CB-Dock2 was used to simulate the interaction between the top four candidate drugs and the corresponding gene-encoded proteins, and the binding energy of each binding site interaction was generated. The effective docking results of the top eight proteins with drugs were shown in Figure 8 and Table 2. CCNA2 showed the lowest binding energy (−11.00 kcal/mol) with irinotecan hydrochloride, indicating that the binding was extremely stable.

**TABLE 2:** Docking results of available proteins with small molecular ligands.

Target	PDB ID	Drug	PubChem ID	Binding energy
CCNA2	7b5l	Irinotecan hydrochloride CTD 00002224	74990	−11
PKD2L1	6du8	Irinotecan hydrochloride CTD 00002224	74990	−9.7
CYP21A2	4y8w	simvastatin CTD 00007319	54454	−9.6
NCSTN	5a63	Isotretinoin PC3 UP	5282379	−9.2
APOM	2wew	Irinotecan hydrochloride CTD 00002224	74990	−8.4
PKD2L1	6du8	Isotretinoin PC3 UP	5282379	−8.2
CCR1	7vl8	simvastatin CTD 00007319	54454	−8.1
APOM	2wew	ciglitazone CTD 00001835	2,750	−8

## 4 Discussion

In this research, a comprehensive SMR analysis was conducted to investigate the druggable gene linked to LSCC, utilizing a combination of GWAS datasets, pharmacogenomic information, and gene expression data (eQTL). We identified ten important drug target genes of LSCC. Among them, COPA, PKD2L1, CCR1, C2, CYP21A2, and NCSTN were risk factors, while CCNA2, C4A, APOM, and LPAR2 were protective factors.

The etiology of LSCC is intricate, encompassing dysregulation of multiple genes and signaling pathways as well as aberrant modulation of cellular processes. Among the risk factors, the tumor-promoting gene COPA (coatomer protein subunit alpha) plays a crucial role in vesicle trafficking within the Golgi apparatus and retrograde transport of cargo proteins between the endoplasmic reticulum (ER) and Golgi, potentially influencing the autoinflammatory process by modulating type I interferon signaling, which has been involved in the pathophysiology of lung cancer (Lepelley et al., 2020; Bao et al., 2022). Furthermore, APOM plays a key role in lipid transport and is implicated in the pathogenesis of emphysema through its association with HDL cholesterol, which has also been linked to lung cancer (Burkart et al., 2014). Additionally, NCSTN acts as an upstream regulator of beta-catenin, facilitating its nuclear translocation and subsequently inducing the ZEB1-mediated epithelial-mesenchymal transition (EMT) process. This EMT process contributes to the acquisition of a malignant phenotype and influences tumor progression (Li et al., 2020). The CC chemokine receptor 1 (CCR1) is crucial in facilitating the recruitment of leukocytes to sites of inflammation (Broxmeyer et al., 1999). The process of tumor invasion and metastasis exhibits numerous similarities with leukocyte trafficking, a phenomenon that is tightly regulated by chemokines and their corresponding receptors. Previous studies have demonstrated a positive correlation between the expression of CCR1 and the aggressive phenotype of NSCLC cells (Wang et al., 2009). Knockdown of CCR1 significantly attenuated the invasive potential of NSCLC cells. The APOM has been identified as a protective factor against the occurrence and progression of various cancers, exhibiting inhibitory actions on cancer cell proliferation, migration, and invasion (Wang et al., 2009; Hu et al., 2015; Zhou et al., 2022; Xu et al., 2023). Lower expression of the C4A gene might be involved in the lung cancer development because of abnormal inflammatory response (Rosenberger et al., 2017).

In order to enhance understanding of the potential pleiotropic effects of the target genes and the potential side effects of the LSCC associated drug, we conducted a comprehensive pharmacability assessment of the therapeutic target potential. Remarkably, five out of ten of the drug target genes investigated in this study have already been analyzed in previous drug development studies (APOM, C4A, CCNA2, COPA, and PKD2L1). However, few research was performed to analyze the adverse reactions of these drug target genes in LSCC treatment, which was adverse to their clinical utilizations. For this reason, we employed a PheWAS analysis to infer potential adverse reactions linked to the intended drug target. Furthermore, enrichment analysis and PPI networks were performed to gain insights into the biological significance underlying these promising drug targets. Finally, drug prediction and molecular docking studies were carried out to further investigate these targets, revealing that a total of four drugs investigated in this study may hold potential clinical significance for the treatment of LSCC by targeting different genes. This further validates the therapeutic value of these target genes as potential drug candidates. Notably, we observed a pronounced affinity of irinotecan towards some genes (CCNA2, PKD2L1, and APOM). Previous studies have reported the therapeutic potential of irinotecan in treating LSCC (Oshita et al., 2011; Wu et al., 2013). Our study provides microscopic evidence to validate the efficacy of irinotecan, thereby offering a theoretical foundation for further elucidation of its underlying mechanism.

The present study has several notable strengths. Initially, owing to the substantial sample sizes in both the MR analysis and population-based studies, our study possesses exceptional statistical power and yields significant findings that may contribute to a deeper understanding of causality. Additionally, we employed HEIDI tests and co-localization methods to mitigate the potential influence of pleiotropy, thereby reducing the likelihood of false positive results. Furthermore, five out of ten identified drug targets have been used in drug development, but the remaining genes still show promise for treating LSCC. The findings suggest that the ten drug target genes identified in the study had significant potential for clinical applications. Nonetheless, we provide a comprehensive list of potential drugs for further testing and research. Finally, insights into the potential causative role of drug target genes on LSCC are provided through additional evidence from single-cell type expression analysis, PPI network, and chemogenic evaluation, thereby further prioritizing potential drug targets.

The present study is subject to certain limitations. Firstly, although MR provides insights into causality, it may not fully replicate real-world clinical trial conditions due to its assumptions about low-dose drug exposure and linear exposure-outcome relationships. This could lead to findings that do not accurately reflect the effects of a drug in a clinical setting (Wu et al., 2013). Future research should aim to connect MR analysis with real-world clinical trials by combining high-dose short-term exposure experiments with MR analysis. In addition, despite efforts to reduce bias, the MR analysis is still susceptible to unmeasured confounding factors or pleiotropy that could affect outcomes (Sanderson, 2021). Thirdly, the lack of direct evidence for the links between certain genes and lung cancer suggests further research is needed to understand how these genes contribute to the development and progression of the disease. Including more omics data and environmental factors in future studies could improve our understanding of the mechanisms of LSCC. Besides, the generalizability of the study is limited by its mostly European sample, requiring more research for broader applicability across ethnicities. Ultimately, the precision of molecular docking analysis depends on the quality of the protein structure and ligand, impacting its ability to identify drug targets but not necessarily their clinical effectiveness (Ballante et al., 2021). Therefore, further investigation and clinical trials are needed to validate the therapeutic viability of these targets and assess their efficacy and safety in practical clinical scenarios.

## Data Availability

The original contributions presented in the study are included in the article/Supplementary Material, further inquiries can be directed to the corresponding author.
