# Supplementary material for: Unveiling potential drug targets for lung squamous cell carcinoma through the integration of druggable genome and genome-wide association data
Source: Front Genet. 2024 Aug 8;15:1431684. doi: 10.3389/fgene.2024.1431684 (PMC11338847; doi:10.3389/fgene.2024.1431684)
Supplement: Supplementary file 1 [file Table2.DOCX]

Supplementary Material

# Supplementary Data

Supplementary Material should be uploaded separately on submission. Please include any supplementary data, figures and/or tables.

Supplementary material is not typeset so please ensure that all information is clearly presented, the appropriate caption is included in the file and not in the manuscript, and that the style conforms to the rest of the article.

# Supplementary Figures and Tables

For more information on Supplementary Material and for details on the different file types accepted, please see [here](https://www.frontiersin.org/guidelines/author-guidelines#supplementary-material).

## Supplementary Figures

**
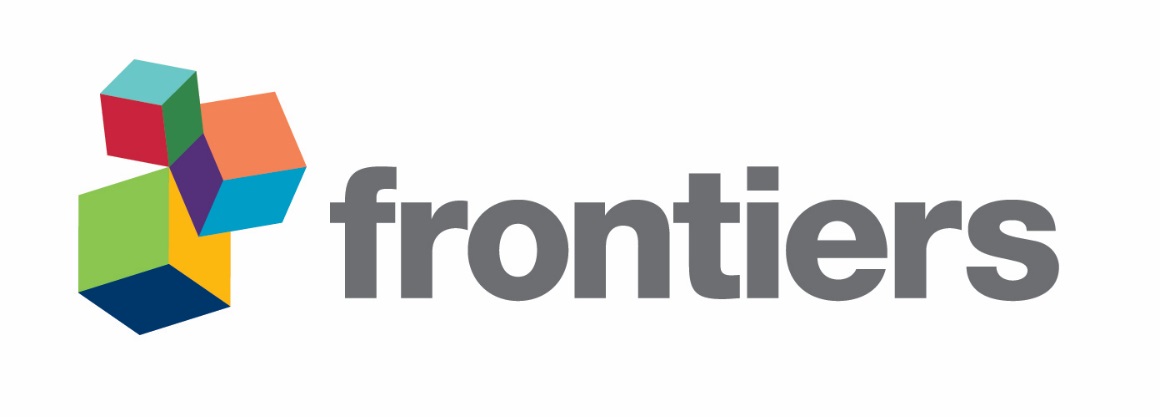
**

Supplementary Figure 1-12. PheWAS results for each gene.

Supplementary Figure 1-12. Results of PheWAS analysis for 6 genes. The bottom dashed line represents the Suggestive line and the top dashed line is the Significant line. Traits that exceeded the significant line were considered to be significantly associated with a gene. As can be seen, there were no significantly associated traits for any of the genes except CCNA2, LPAR2 and NCSTN, and information on traits significantly associated with CCNA2, LPAR2 and NCSTN is shown in Supplementary table S8.


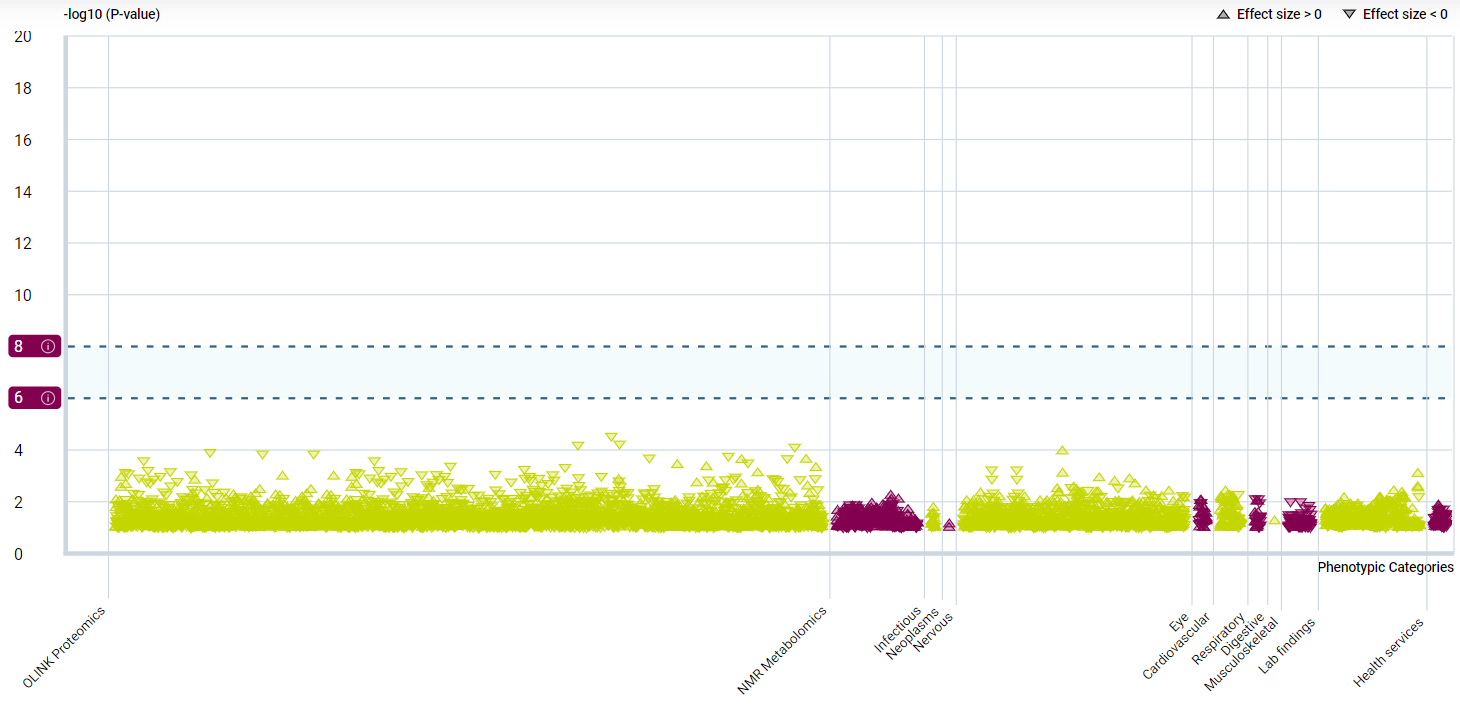


**Supplementary Figure 1.** Continuous traits PheWAS association with APOM.


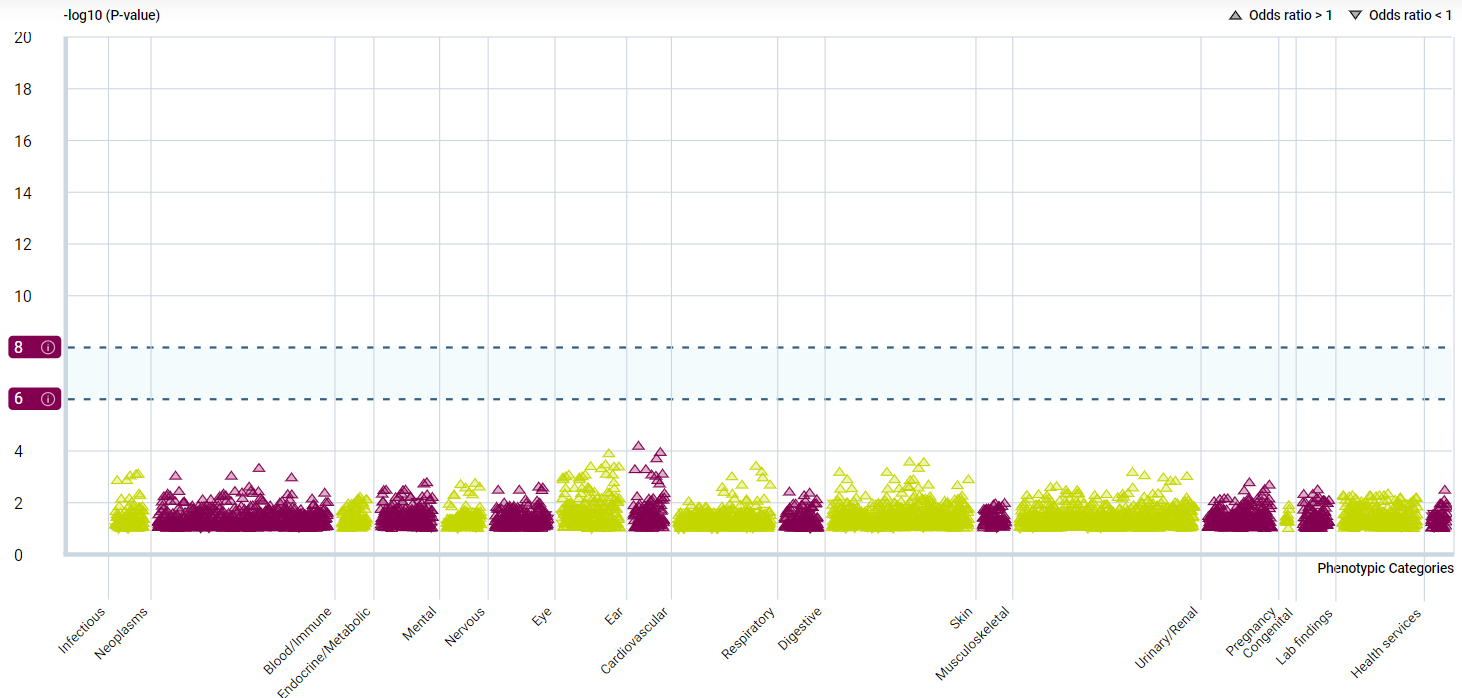


**Supplementary Figure 2.** Binary traits PheWAS association with APOM.


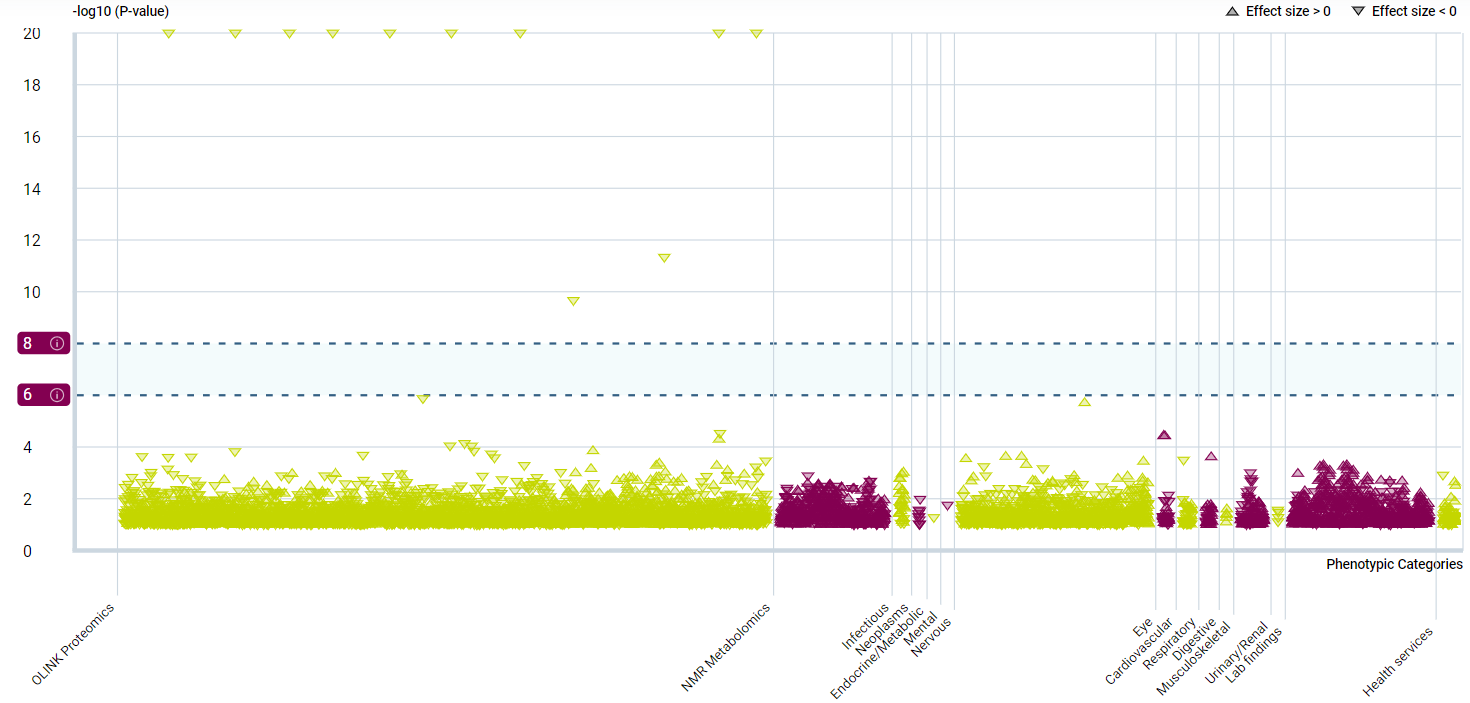


**Supplementary Figure 3.** Continuous traits PheWAS association with C2.


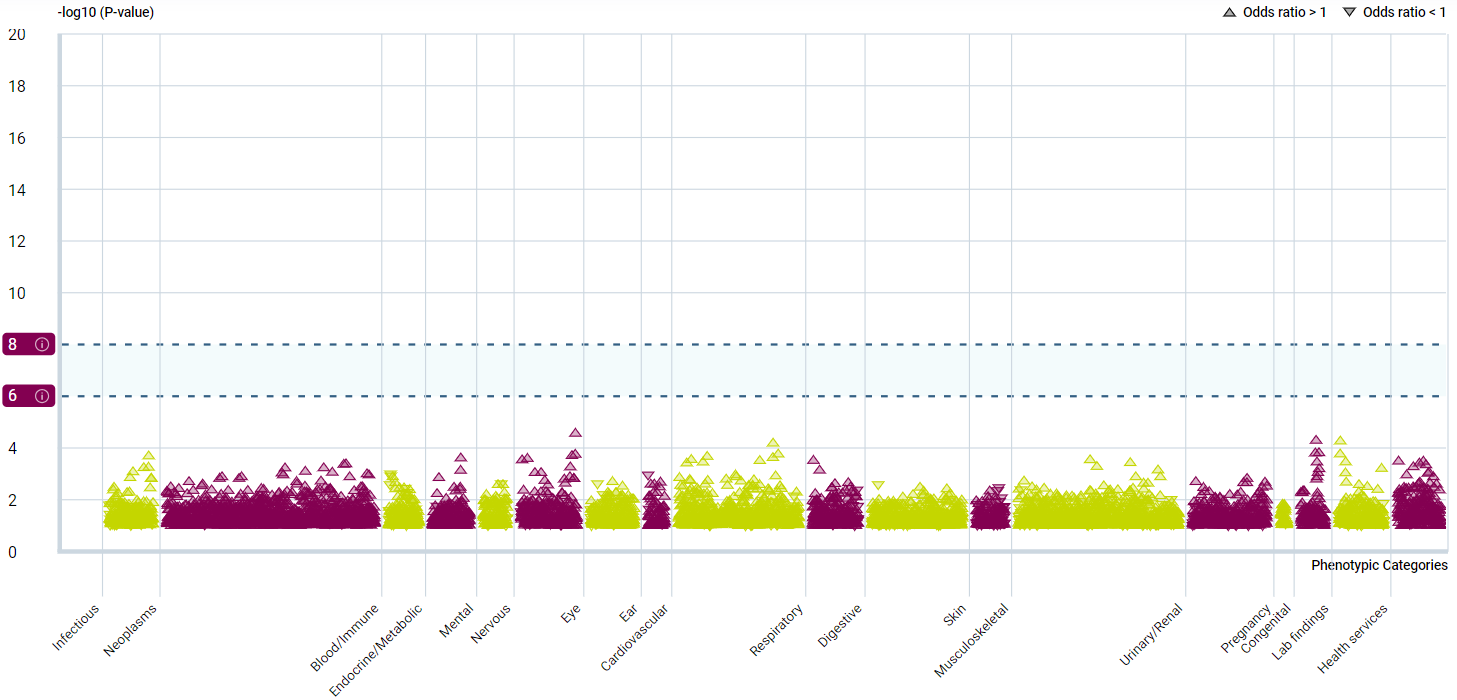


**Supplementary Figure 4.** Binary traits PheWAS association with C2.


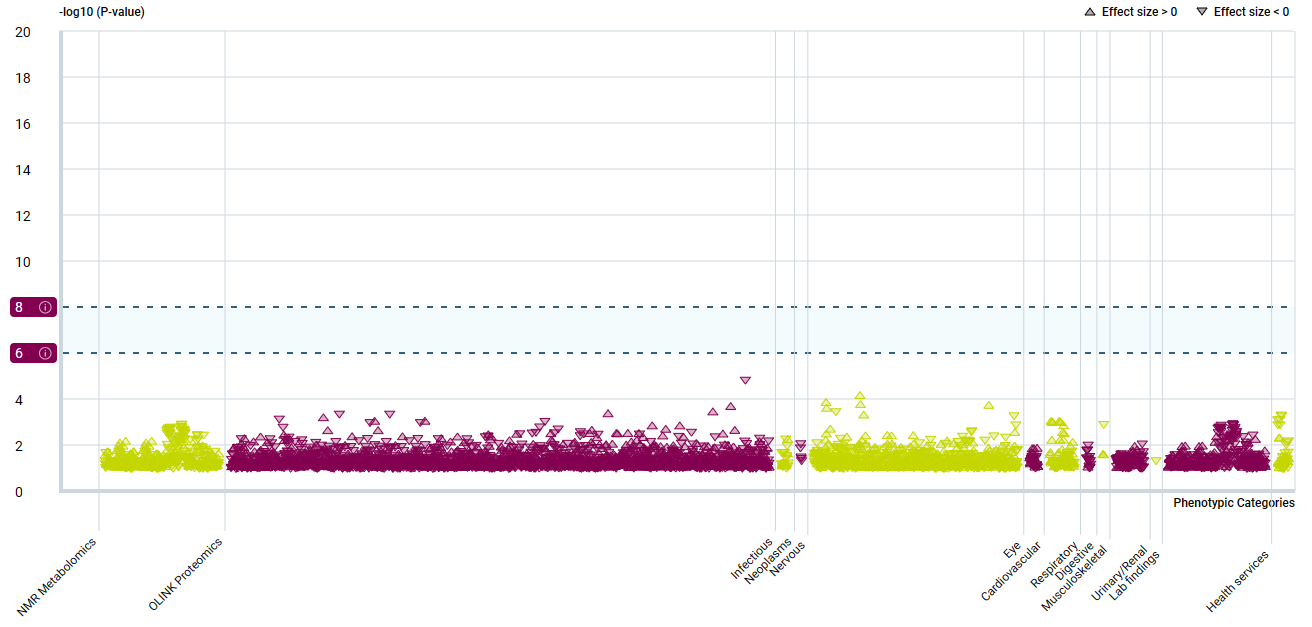


**Supplementary Figure 5.** Continuous traits PheWAS association with CCR1.


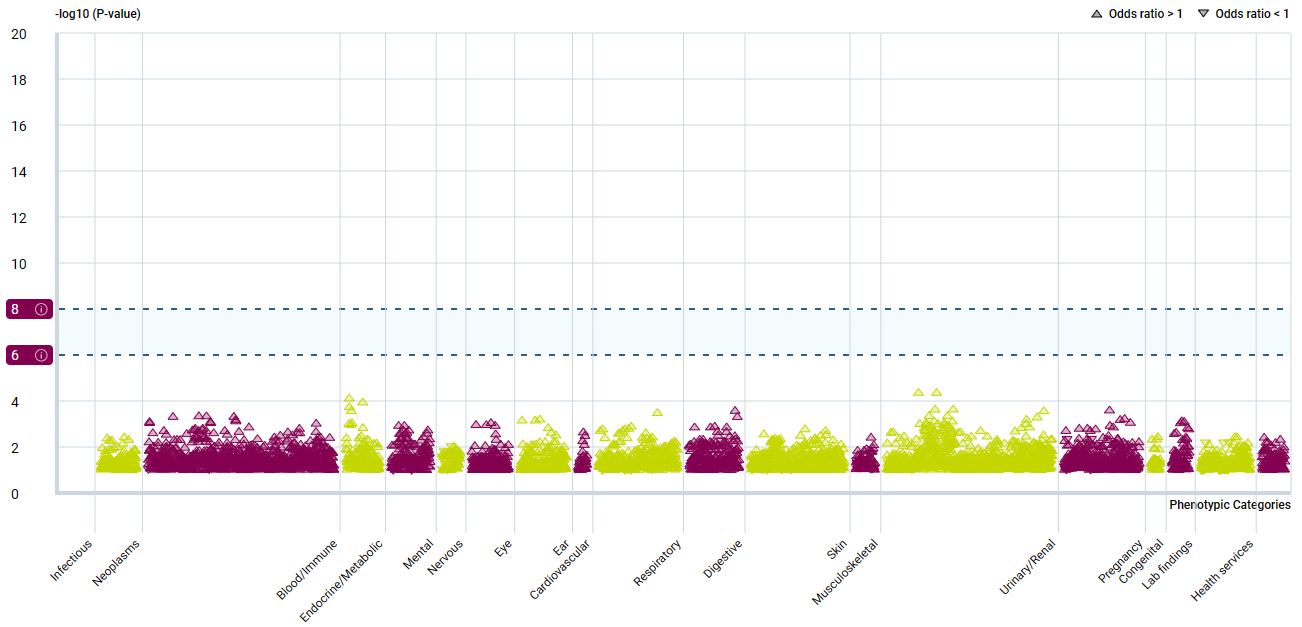


**Supplementary Figure 6.** Binary traits PheWAS association with CCR1.


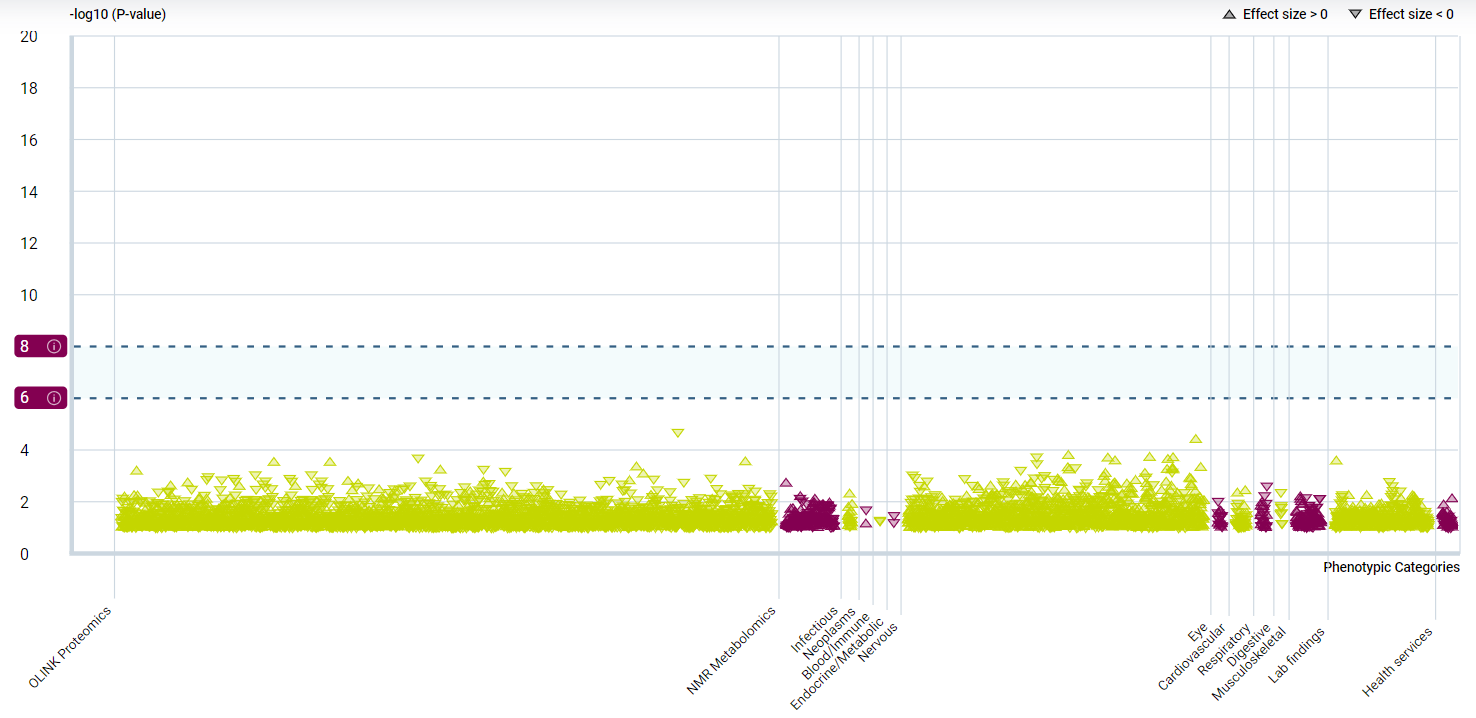


**Supplementary Figure 7.** Continuous traits PheWAS association with COPA.


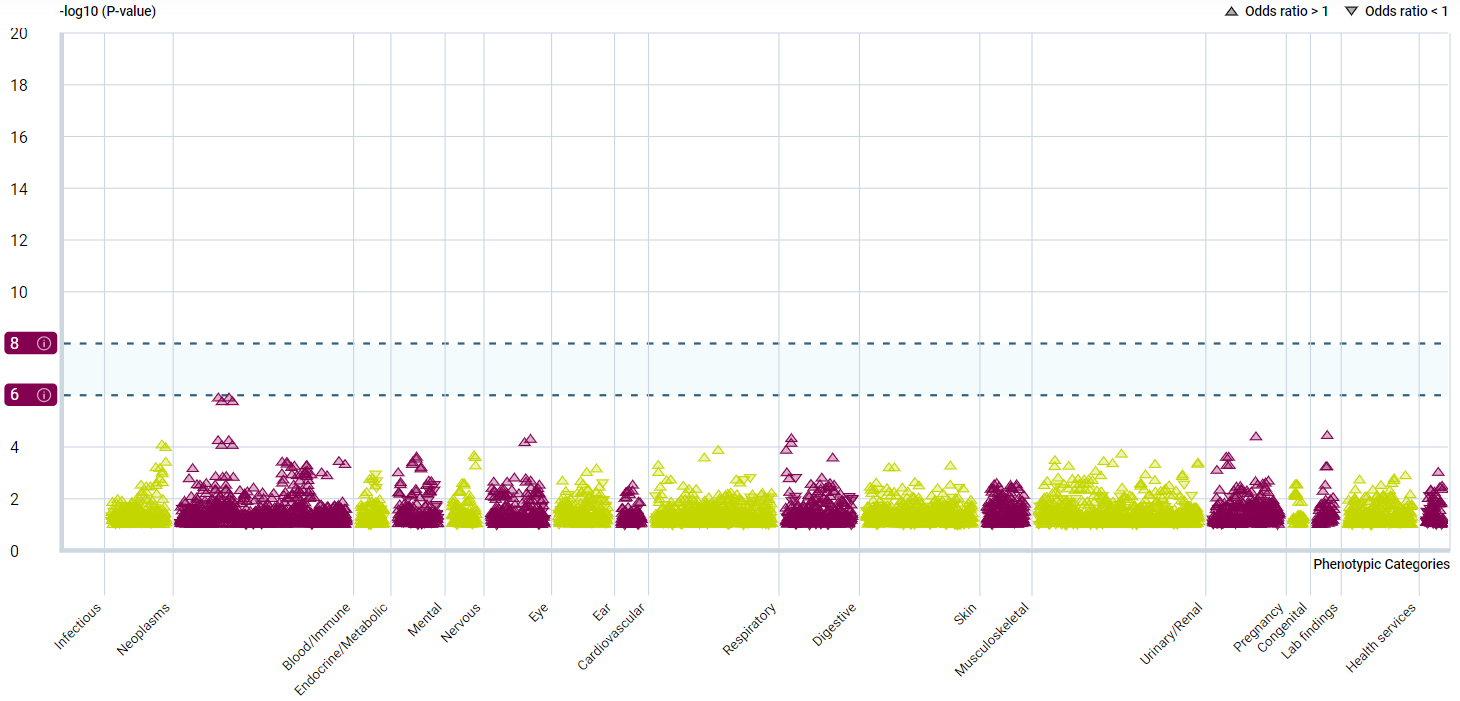


**Supplementary Figure 8.** Binary traits PheWAS association with COPA.


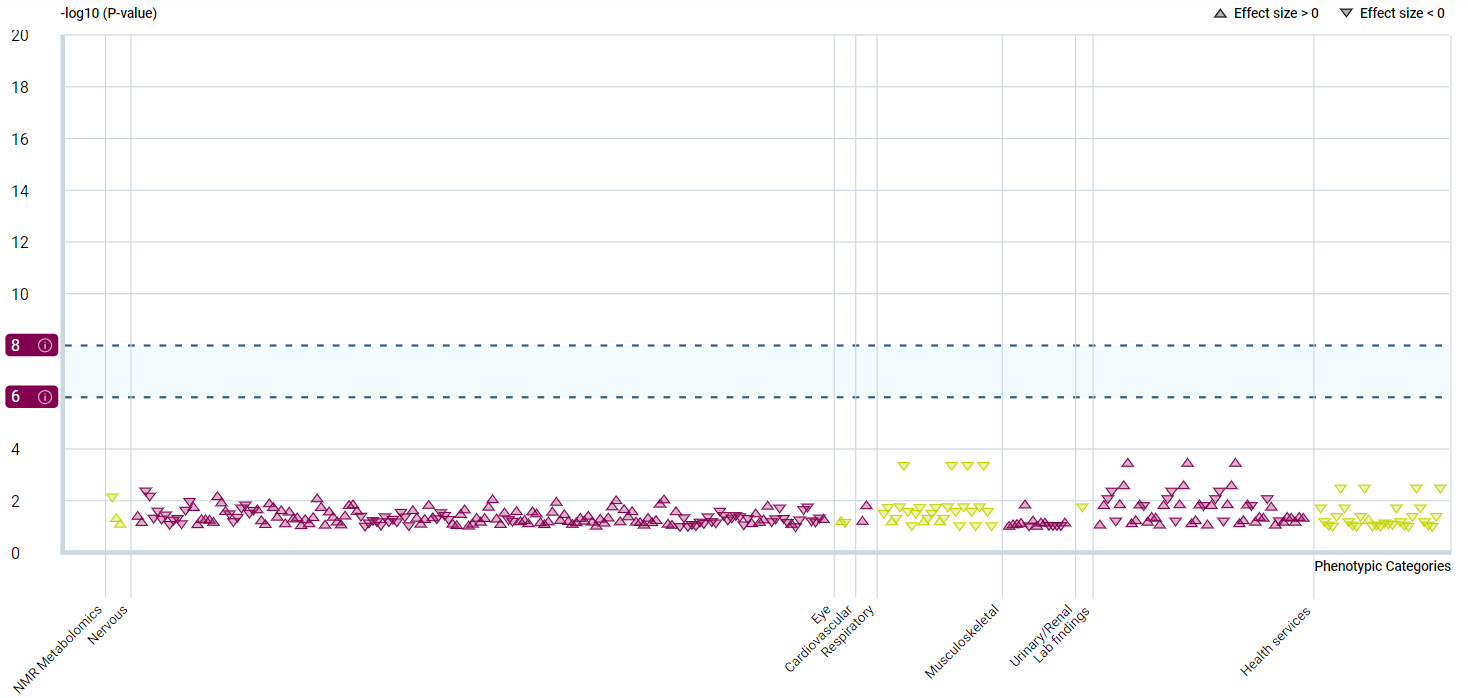


**Supplementary Figure 9.** Continuous traits PheWAS association with CYP21A2.


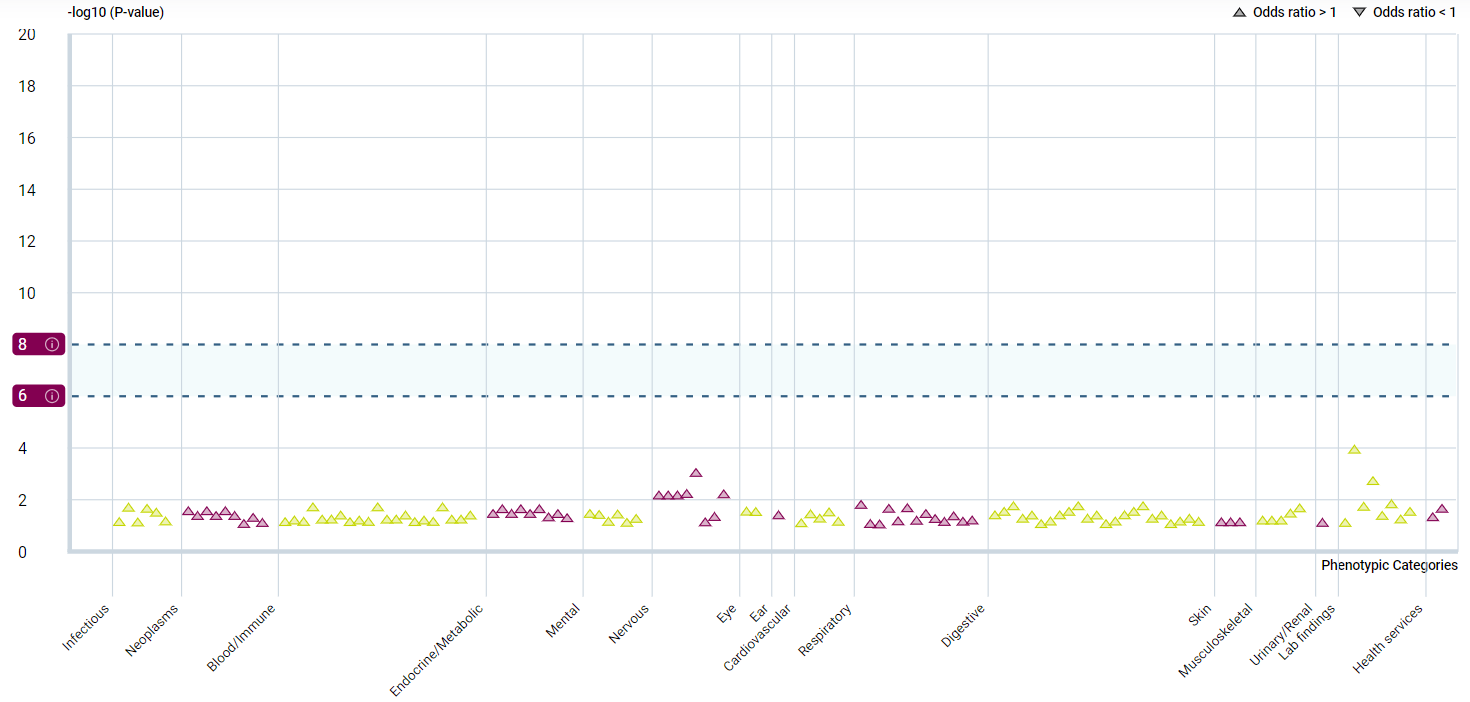


**Supplementary Figure 10.** Binary traits PheWAS association with CYP21A2.


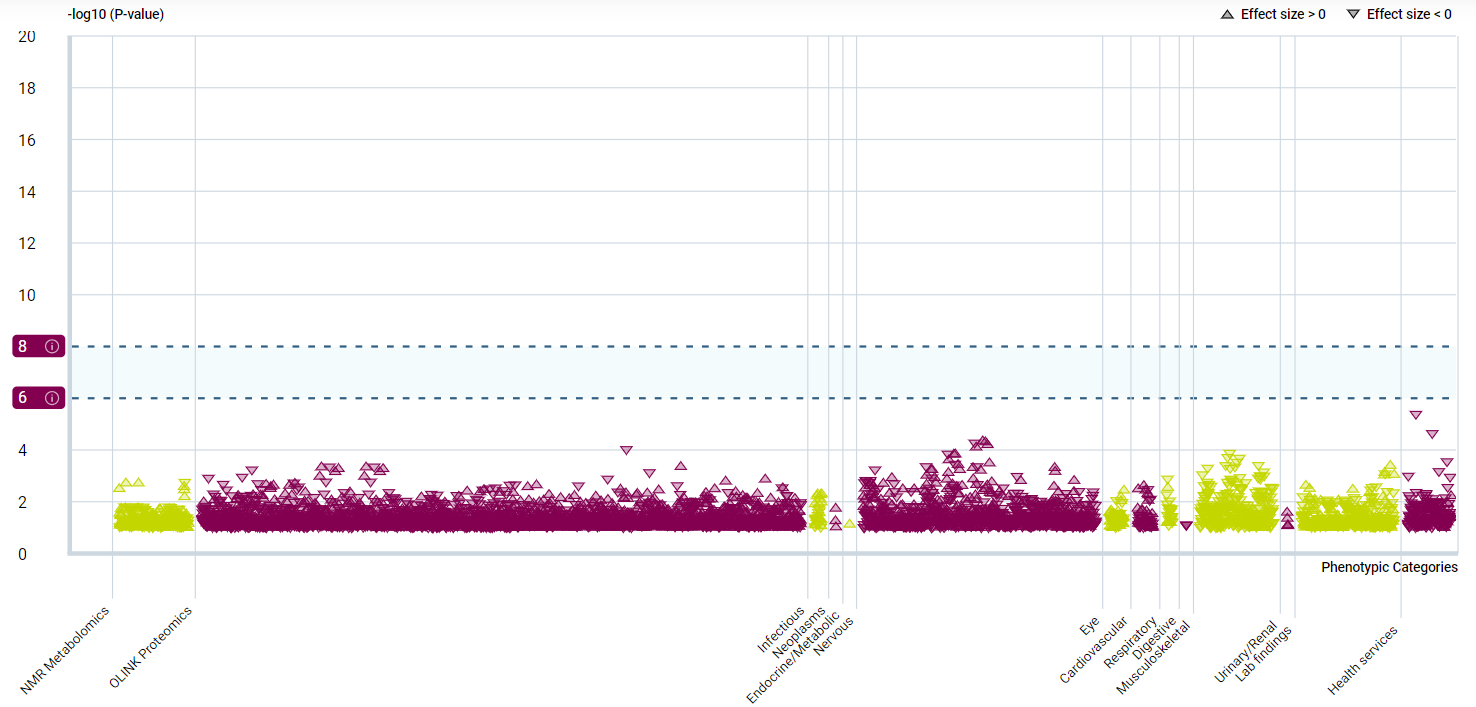


**Supplementary Figure 11.** Continuous traits PheWAS association with PKD2L1.


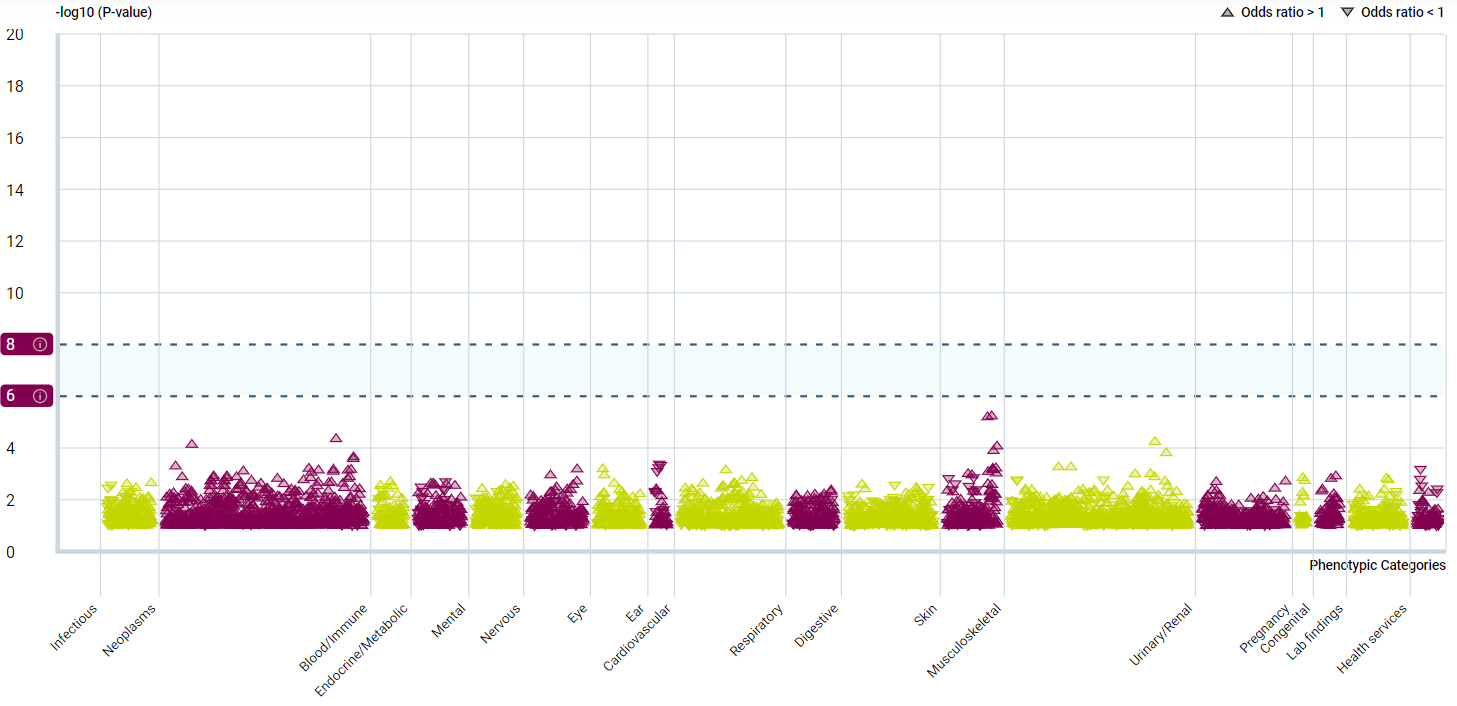


**Supplementary Figure 12.** Binary traits PheWAS association with PKD2L1.
